# Supplementary material for: A meta-analysis of the prevalence, genotype distribution and risk factors for human papillomavirus infection in Nepal
Source: PLoS One. 2025 Sep 12;20(9):e0332214. doi: 10.1371/journal.pone.0332214 (PMC12431021; doi:10.1371/journal.pone.0332214)
Supplement: S1 Appendix — (DOCX) [file pone.0332214.s001.docx]

# For PubMed:

#1: (((("Papillomaviridae"[Mesh]) OR "Human Papillomavirus Viruses"[Mesh]) OR "Human papillomavirus 16"[Mesh]) OR "Human papillomavirus 18"[Mesh]) OR "Papillomavirus Infections"[Mesh]

#2: (("papillomaviridae"[tiab] OR "human papillomavirus"[tiab] OR "hpv"[tiab] OR "human papillomavirus viruses"[tiab]) OR ("human papillomavirus 16"[tiab] OR "hpv-16"[tiab] OR "hpv 16"[tiab]) OR ("human papillomavirus 18"[tiab] OR "hpv-18"[tiab] OR "hpv 18"[tiab]) OR ("papillomavirus infections"[tiab] OR "hpv infection"[tiab]))

#3: (("nepal"[tiab] OR "nepali"[tiab]) OR ("south asia"[tiab] AND ("hpv"[tiab] OR "papillomavirus"[tiab])))

#4: #1 OR #2 AND #3

# For Embase:

#1: ('papillomaviridae'/exp OR 'human papillomavirus viruses'/exp OR 'human papillomavirus 16'/exp OR 'human papillomavirus 18'/exp OR 'papillomavirus infections'/exp)

#2: ('papillomaviridae':ti,ab OR 'human papillomavirus':ti,ab OR 'hpv':ti,ab OR 'human papillomavirus viruses':ti,ab OR 'human papillomavirus 16':ti,ab OR 'hpv-16':ti,ab OR 'hpv 16':ti,ab OR 'human papillomavirus 18':ti,ab OR 'hpv-18':ti,ab OR 'hpv 18':ti,ab OR 'papillomavirus infections':ti,ab OR 'hpv infection':ti,ab)

#3: ('nepal':ti,ab OR 'nepali':ti,ab OR ('south asia':ti,ab AND ('hpv':ti,ab OR 'papillomavirus':ti,ab)))

#4: #1 OR #2

#5: #4 AND #3

# For Google Scholar:

("human papillomavirus" OR "hpv" OR "papillomavirus infection" OR "hpv 16" OR "hpv 18")

AND ("prevalence" OR "epidemiology" OR "genotype" OR "risk factors")

AND ("Nepal" OR "South Asia")
