# Supplementary material for: A meta-analysis of the prevalence, genotype distribution and risk factors for human papillomavirus infection in Nepal
Source: PLoS One. 2025 Sep 12;20(9):e0332214. doi: 10.1371/journal.pone.0332214 (PMC12431021; doi:10.1371/journal.pone.0332214)
Supplement: S2 Appendix — (DOCX) [file pone.0332214.s002.docx]

| Author/ Year | | Mean age | Age at first marriage | Sampe Size | No.of cases | Genotype 16 | Genotype 18 | Smoking status of HPV infected | Educational status of HPV infected | HPV infected participant’s multiple marriage/ sexual partners | HPV infected participant's Husband’s multiple marriage / sexual partners | HPV cases with STI | HPV patients with contraceptives use | Husband’s migration status of HPV patients |
| --- | --- | --- | --- | --- | --- | --- | --- | --- | --- | --- | --- | --- | --- | --- |
| Thapa et al. 2018 | 32.6 ± 8.6 | 16.7 ± 3.8 | 998 | 115 | 67 | 12 | 14/158 | 66/588 | 5/70 | 23/118 | 22/148 | 5/95 |  |  |
| Derek et al. 2014 | 33.8 ± 8.8 | 17.3 ± 2.6 | 261 | 25 | 5 |  |  |  |  |  |  |  |  |  |
| Sherpa et al. 2010 | 34.68 ± 12.05 | 17 ± 2.96 | 932 | 80 | 18 | 5 |  | 34/508 | 11/91 | 31/278 |  |  |  |  |
| Shakya et al. 1 2018 | 40 | 18 | 1498 | 214 |  |  |  |  |  |  |  |  |  |  |
| Shakya et al. 2 2016 | 40 | 18 | 1289 | 102 | 10 | 30 | 34/306 | 23/207 | 6/44 | 20/145 |  |  |  |  |
| Bhatta et al. 2017 | 39.5 ± 8.1 | 19.2 ± 4.0 | 542 | 46 |  |  | 2/56 | 42/456 | 1/14 | 6/67 |  |  | 10/111 |  |
| Shrestha et al. 2023 | 31.17±5.57 |  | 199 | 6 | 4 | 2 |  |  |  |  |  |  |  |  |
| Johnson et al. 2015 | 33.9 ± 8.8 |  | 363 | 20 |  |  |  |  |  |  |  | 13/150 | 10/110 |  |
