# Supplementary material for: A meta-analysis of the prevalence, genotype distribution and risk factors for human papillomavirus infection in Nepal
Source: PLoS One. 2025 Sep 12;20(9):e0332214. doi: 10.1371/journal.pone.0332214 (PMC12431021; doi:10.1371/journal.pone.0332214)
Supplement: S3 Appendix — (DOCX) [file pone.0332214.s003.docx]

Quality assessment of included studies

| Study | Domain | Judgement | Score | Reasoning |
| --- | --- | --- | --- | --- |
| **Thapa et al. 2018** | Source of Information | Yes | 1 | Data were collected through mobile screening clinics; data collection procedures were clearly described. |
|  | Inclusion/Exclusion Criteria | Yes | 1 | Explicit criteria: married, non-pregnant, aged 20–65, apparently healthy, no history of cervical cancer. |
|  | Time Period Identified | Yes | 1 | Study period clearly mentioned: May 2016 – January 2017. |
|  | Consecutive Subjects Indicated | No | 0 | Participants were selected using purposive sampling; not consecutive or randomly selected. |
|  | Evaluator Masking | No | 0 | No mention of blinding in cytology reading or PCR/genotyping analysis. |
|  | Quality Assurance Measures | Yes | 1 | Standardized protocols followed (e.g., WHO guidelines, DNA quality checked, ethical approval obtained). |
|  | Exclusion from Analysis | Yes | 1 | 52 excluded samples clearly explained and documented (not suitable for analysis). |
|  | Confounding Control | Yes | 1 | Statistical analysis accounted for multiple confounders (e.g., age, parity, sexual behavior, HIV status). |
|  | Missing Data Handling | Unclear | 0 | No specific mention of how missing data in socio-demographics or cytology were handled. |
|  | Response Rates/Data Completeness | No | 0 | Response rate not reported; unclear how many women declined or were eligible but not included. |
|  | Follow-Up Details | Not Applicable | 0 | No follow-up planned or conducted; cross-sectional design. |
| **Total Score** |  |  | **6** | **Moderate Quality** |
|  |  |  |  |  |
| **Johnson et al. 2014** | Source of Information | Yes | 1 | Data were collected during a structured health camp with detailed descriptions of sample collection and laboratory testing. |
|  | Inclusion/Exclusion Criteria | Yes | 1 | Criteria included women aged 16–60, non-pregnant, not menstruating, and with a cervix; clearly stated in the methods. |
|  | Time Period Identified | Yes | 1 | The health camp occurred on July 5, 2013; precise date provided. |
|  | Consecutive Subjects Indicated | No | 0 | Recruitment was via a single-day health camp with voluntary attendance; not population-based or consecutive sampling. |
|  | Evaluator Masking | No | 0 | No mention of blinding of cytology readers or lab personnel to clinical/demographic data or sample type. |
|  | Quality Assurance Measures | Yes | 1 | Sample handling and testing procedures followed standardized APTIMA protocols; translated and back-translated surveys indicate QA effort. |
|  | Exclusion from Analysis | Yes | 1 | Exclusions (e.g., invalid samples, lack of matching samples) were explained and documented. |
|  | Confounding Control | Yes | 1 | Data analysis included stratified Kappa statistics and demographic analysis, although no regression models were used. |
|  | Missing Data Handling | Unclear | 0 | Missing data (e.g., incomplete paired samples and cytology results) acknowledged but not analytically addressed. |
|  | Response Rates/Data Completeness | No | 0 | Total registered vs. enrolled women provided, but no formal response rate or discussion of participation bias. |
|  | Follow-Up Details | Not Applicable | 0 | Cross-sectional design with no longitudinal follow-up; referrals were noted, but no outcome follow-up was tracked. |
| **Total Score** |  |  | **6** | **Moderate Quality** |
|  |  |  |  |  |
| **Sherpa et al. 2010** | Source of Information | Yes | 1 | Data from a population-based study using home visits and clinic exams; sample collection and lab methods clearly described. |
|  | Inclusion/Exclusion Criteria | Yes | 1 | All women aged 15–59 were eligible; final sample limited to married women due to consent issues. |
|  | Time Period Identified | Yes | 1 | October 2006 to March 2007 reported. |
|  | Consecutive Subjects Indicated | No | 0 | Voluntary participation with high refusal rate; not a consecutive or random sample. |
|  | Evaluator Masking | No | 0 | No mention of blinding for cytology or histology examiners. |
|  | Quality Assurance Measures | Yes | 1 | Ethical approvals, validated lab methods, and quality control measures (e.g., beta-globin testing) included. |
|  | Exclusion from Analysis | Yes | 1 | Samples with inadequate DNA or cytology excluded and documented. |
|  | Confounding Control | Yes | 1 | Adjusted ORs provided for demographic and behavioral factors. |
|  | Missing Data Handling | Yes | 1 | Detailed reporting of exclusions due to missing or invalid data. |
|  | Response Rates/Data Completeness | Yes | 1 | Response rates and reasons for non-participation discussed clearly. |
|  | Follow-Up Details | Not Applicable | 0 | Cross-sectional study with no follow-up planned. |
| **Total Score** |  |  | **8** | **High Quality** |
|  |  |  |  |  |
| **Shakya et al. 2018** | Source of Information | Yes | 1 | Detailed methods for clinical and lab sample collection; well-described STI and HPV testing. |
|  | Inclusion/Exclusion Criteria | Yes | 1 | Married, non-pregnant women ≥15 years old; clear reasons for exclusions documented. |
|  | Time Period Identified | Yes | 1 | February 2012 to May 2013 noted explicitly. |
|  | Consecutive Subjects Indicated | No | 0 | Voluntary participation through village-based invitation; no consecutive sampling. |
|  | Evaluator Masking | No | 0 | No blinding of lab or clinical evaluators is described. |
|  | Quality Assurance Measures | Yes | 1 | Samples transported on ice; internal controls in PCR; ethical approvals obtained. |
|  | Exclusion from Analysis | Yes | 1 | Incomplete or inadequate samples excluded with documentation. |
|  | Confounding Control | Yes | 1 | Multivariate logistic regression used to assess associations (e.g., trichomoniasis and demographics). |
|  | Missing Data Handling | Yes | 1 | Detailed tracking of sample losses and lab testing gaps; flow chart provided. |
|  | Response Rates/Data Completeness | Yes | 1 | 62% participation rate reported; missing samples and refusals detailed. |
|  | Follow-Up Details | Not Applicable | 0 | Cross-sectional with no follow-up; lab testing only. |
| **Total Score** |  |  | **8** | **High Quality** |
|  |  |  |  |  |
| **Shakya et al.** | Source of Information | Yes | 1 | Population-based sampling; detailed lab and questionnaire methods. |
|  | Inclusion/Exclusion Criteria | Yes | 1 | Non-pregnant married women ≥15 years included; exclusions clearly stated. |
|  | Time Period Identified | Yes | 1 | Conducted February 2012–May 2013. |
|  | Consecutive Subjects Indicated | No | 0 | Voluntary enrollment through FCHVs; no randomized or consecutive design. |
|  | Evaluator Masking | No | 0 | No blinding mentioned for cytology or PCR analysis. |
|  | Quality Assurance Measures | Yes | 1 | Internal PCR controls, pathologist review, and international lab partnerships included. |
|  | Exclusion from Analysis | Yes | 1 | Invalid results and inadequate samples were excluded and described. |
|  | Confounding Control | Yes | 1 | Logistic regression adjusted for age, education, marriage history, etc. |
|  | Missing Data Handling | Yes | 1 | Data loss and missing tests documented; only valid samples analyzed. |
|  | Response Rates/Data Completeness | Yes | 1 | 62% response rate; reasons for refusal and missing data clearly reported. |
|  | Follow-Up Details | Not Applicable | 0 | No follow-up conducted as part of the study design. |
| **Total Score** |  |  | **8** | **High Quality** |
|  |  |  |  |  |
| Bhatta et al. 2017 | Source of Information | Yes | 1 | Data from a structured women's health camp; standardized lab testing and cytology protocols detailed. |
|  | Inclusion/Exclusion Criteria | Yes | 1 | Inclusion: women >18, not pregnant/menstruating, with a cervix; exclusion criteria and consent procedures clearly outlined. |
|  | Time Period Identified | Yes | 1 | Study conducted in 2014, clearly stated. |
|  | Consecutive Subjects Indicated | No | 0 | Convenience sample from a single health camp; no indication of consecutive or random sampling. |
|  | Evaluator Masking | No | 0 | No mention of blinding for cytology or HPV test evaluators. |
|  | Quality Assurance Measures | Yes | 1 | International lab collaboration; standard protocols (APTIMA, Bethesda); ethical clearance obtained. |
|  | Exclusion from Analysis | Yes | 1 | Non-usable or incomplete samples were excluded with explanation. |
|  | Confounding Control | Yes | 1 | Multivariable logistic regression conducted for key predictors (age, education, spousal migration). |
|  | Missing Data Handling | Yes | 1 | Clear reporting of missing data with footnotes in tables. |
|  | Response Rates/Data Completeness | No | 0 | Convenience sample with no formal report of response rates or recruitment denominator. |
|  | Follow-Up Details | Not Applicable | 0 | Cross-sectional design; no follow-up involved. |
| **Total Score** |  |  | **7** | **Moderate Quality** |
|  |  |  |  |  |
| **Shrestha et al. 2023** | Source of Information | Yes | 1 | Samples collected by gynecologic oncologists at a tertiary care hospital with clear lab processing protocols. |
|  | Inclusion/Exclusion Criteria | Yes | 1 | Inclusion/exclusion well stated (women >18 with consent, complete data only); ethics clearance obtained. |
|  | Time Period Identified | Yes | 1 | Study conducted between June and November 2022. |
|  | Consecutive Subjects Indicated | No | 0 | Convenience sampling used; not population-based or consecutive. |
|  | Evaluator Masking | No | 0 | No mention of blinding in cytology or molecular lab procedures. |
|  | Quality Assurance Measures | Yes | 1 | Good lab protocol described; Bethesda system used; follow-up confirmation attempted via contact. |
|  | Exclusion from Analysis | Yes | 1 | Incomplete records and those without consent were excluded; flow process clarified. |
|  | Confounding Control | No | 0 | Only descriptive statistics reported; no regression or control for confounding variables. |
|  | Missing Data Handling | Yes | 1 | Ambiguous/missing records clarified through healthcare contact; exclusion process described. |
|  | Response Rates/Data Completeness | No | 0 | No data on eligible participants or formal response rate reported. |
|  | Follow-Up Details | Not Applicable | 0 | No longitudinal follow-up conducted. |
| **Total Score** |  |  | **6** | **Moderate Quality** |
|  |  |  |  |  |
| **Johnson et al. 2016** | Source of Information | Yes | 1 | Data from RH camp, lab testing in US (Hologic/Gen-Probe), survey well described. |
|  | Inclusion/Exclusion Criteria | Yes | 1 | Clear inclusion: married women, aged 16–60, not menstruating or pregnant; informed consent obtained. |
|  | Time Period Identified | Yes | 1 | Study conducted in April 2014; clearly stated. |
|  | Consecutive Subjects Indicated | No | 0 | Participants recruited from a single RH camp; no population-based or consecutive sampling. |
|  | Evaluator Masking | No | 0 | No mention of evaluator blinding for cytology or HPV analysis. |
|  | Quality Assurance Measures | Yes | 1 | International lab, Bethesda system, standardized protocols; survey translated and back-translated. |
|  | Exclusion from Analysis | Yes | 1 | Only valid test results included in final analysis; clearly documented. |
|  | Confounding Control | Yes | 1 | Multivariate logistic regression used for both HR-HPV and cytology outcomes; controlled for age, contraception, etc. |
|  | Missing Data Handling | Yes | 1 | Data losses explained in flowchart; missing responses reported per analysis. |
|  | Response Rates/Data Completeness | No | 0 | No formal response rate or number of non-participants described. |
|  | Follow-Up Details | Not Applicable | 0 | No follow-up tracked; cross-sectional design. |
| **Total Score** |  |  | **7** | **Moderate Quality** |
