# Supplementary material for: A meta-analysis of the prevalence, genotype distribution and risk factors for human papillomavirus infection in Nepal
Source: PLoS One. 2025 Sep 12;20(9):e0332214. doi: 10.1371/journal.pone.0332214 (PMC12431021; doi:10.1371/journal.pone.0332214)
Supplement: S6 Appendix — (DOCX) [file pone.0332214.s006.docx]

**S2 Table. Sensitivity Analysis by omitting one study at a time using random effect model**

| Author Year | Incidence (%) | Lower 95% C.I. | Upper 95% C.I. | Tau^2^ | I^2^ (%) |
| --- | --- | --- | --- | --- | --- |
| Omitting Thapa et al. 2018 | 8.22 | 5.60 | 10.8 | 0.0011 | 91.03 |
| Omitting Derek et al. 2014 | 8.52 | 5.8 | 11.2 | 0.0012 | 92.65 |
| Omitting Sherpa et al. 2010 | 8.64 | 5.8 | 11.4 | 0.0013 | 91.83 |
| Omitting Shakya et al. 1 2018 | 7.8 | 5.7 | 9.8 | <0.001 | 84.48 |
| Omitting Shakya et al. 2 2016 | 8.75 | 5.9 | 11.5 | 0.001 | 91.17 |
| Omitting Bhatta et al. 2017 | 8.66 | 5.9 | 11.4 | 0.001 | 92.36 |
| Omitting Shrestha et al. 2023 | 9.44 | 7.3 | 11.6 | <0.001 | 87.06 |
| Omitting Johnson et al. 2015 | 9.08 | 6.5 | 11.7 | <0.001 | 91.21 |
