# Supplementary material for: A meta-analysis of the prevalence, genotype distribution and risk factors for human papillomavirus infection in Nepal
Source: PLoS One. 2025 Sep 12;20(9):e0332214. doi: 10.1371/journal.pone.0332214 (PMC12431021; doi:10.1371/journal.pone.0332214)
Supplement: S7 Appendix — Smoking b. Educational status c. Multiple sexual partners (self) d. Multiple sexual partners of husbands e. History of sexually transmitted infections (STIs) f. Contraceptive use g. Migration status. (PDF) [file pone.0332214.s007.pdf]

Study	Cases Exposed	Controls Exposed	Cases Non-Exposed	Controls Non-Exposed	logOR	95%-CI	Weight (common)	Weight (random)
Thapa et al. 2018	14	144	101	739	-0.34	[-0.93; 0.25]	32.9%	38.9%
Shakya et al. 2016	34	372	68	915	0.21	[-0.22; 0.64]	61.6%	49.0%
Bhatta et al. 2017	2	54	50	500	-0.99	[-2.43; 0.45]	5.5%	12.1%
**Common effect model**					**-0.04**	**[-0.38; 0.30]**	**100.0%**	**.**
**Random effects model (HK)**					**-0.15**	**[-1.38; 1.08]**	**.**	**100.0%**

Heterogeneity: $I^2 = 49.5\%$, $\tau^2 = 0.1137$, $\chi^2_2 = 3.96$ ($p = 0.1380$)

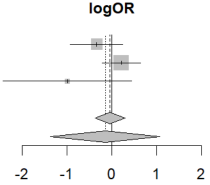

Log(OR) and SE:

Log(OR) = -0.151

SE = 0.285

P-value for pooled Log(OR) (random-effects model): 0.648

Study	Cases Exposed	Controls Exposed	Cases Non-Exposed	Controls Non-Exposed	logOR	95%-CI	Weight (common)	Weight (random)
Thapa et al. 2018	66	522	49	361	-0.07	[-0.46; 0.32]	36.1%	28.5%
Sherpa et al. 2010	34	474	46	378	-0.53	[-0.99; -0.07]	26.0%	25.9%
Shakya et al. 2016	23	184	79	1003	0.46	[-0.03; 0.95]	23.2%	24.9%
Bhatta et al. 2017	42	414	15	170	0.14	[-0.48; 0.76]	14.7%	20.6%
**Common effect model**					**-0.04**	**[-0.27; 0.20]**	**100.0%**	**.**
**Random effects model (HK)**					**-0.01**	**[-0.68; 0.65]**	**.**	**100.0%**

Heterogeneity: $I^2 = 65.3\%$, $\tau^2 = 0.1128$, $\chi^2_3 = 8.64$ (p = 0.0344)

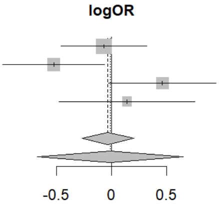

Log(OR) and SE:

Log(OR) = -0.013

SE = 0.209

P-value for pooled Log(OR) (random-effects model): 0.953

Study	Cases Exposed	Controls Exposed	Cases Non-Exposed	Controls Non-Exposed	logOR	95%-CI	Weight (common)	Weight (random)
Thapa et al. 2018	5	65	110	818	-0.56	[-1.49; 0.37]	17.0%	24.6%
Sherpa et al. 2010	11	80	68	760	0.43	[-0.25; 1.11]	32.2%	25.3%
Shakya et al. 2016	6	38	96	1149	0.64	[-0.25; 1.52]	18.8%	24.7%
Bhatta et al. 2017	44	13	54	549	3.54	[2.86; 4.22]	32.0%	25.3%
**Common effect model**					**1.30**	**[0.91; 1.68]**	**100.0%**	**.**
**Random effects model (HK)**					**1.03**	**[-1.79; 3.84]**	**.**	**100.0%**

Heterogeneity: $I^2 = 95.4\%$, $\tau^2 = 3.2929$, $\chi^2_3 = 65.56$ ($p < 0.0001$)

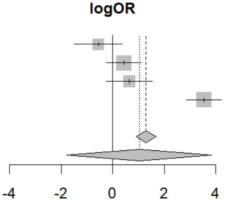

Log(OR) and SE:

Log(OR) = 1.026

SE = 0.884

P-value for pooled Log(OR) (random-effects model): 0.33

Study	Cases Exposed	Controls Exposed	Cases Non-Exposed	Controls Non-Exposed	logOR	95%-CI	Weight (common)	Weight (random)
Thapa et al. 2018	23	95	92	788	0.73	[0.23; 1.23]	30.3%	30.3%
Sherpa et al. 2010	31	247	40	548	0.54	[0.05; 1.03]	31.8%	31.8%
Shakya et al. 2016	20	125	82	1062	0.73	[0.21; 1.25]	28.2%	28.2%
Bhatta et al. 2017	6	61	49	501	0.01	[-0.88; 0.89]	9.8%	9.8%
**Common effect model**					**0.60**	**[0.32; 0.88]**	**100.0%**	**.**
**Random effects model (HK)**					**0.60**	**[0.21; 0.99]**	**.**	**100.0%**

Heterogeneity: $I^2 = 0.0\%$, $\tau^2 = 0$, $\chi^2_3 = 2.26$ ($p = 0.5207$)

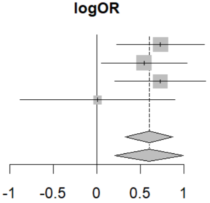

Log(OR) and SE:

Log(OR) = 0.599

SE = 0.123

P-value for pooled Log(OR) (random-effects model): 0.0165

Study	Cases Exposed	Controls Exposed	Cases Non-Exposed	Controls Non-Exposed	logOR	95%-CI
Thapa et al. 2018	22	126	93	757	0.35	[-0.15; 0.85]

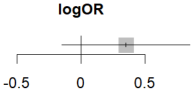

Log(OR) and SE:
Log(OR) = 0.352
SE = 0.256

P-value for pooled Log(OR) (random-effects model): 0.169

Study	Cases Exposed	Controls Exposed	Cases Non-Exposed	Controls Non-Exposed	logOR	95%-CI	Weight (common)	Weight (random)
Thapa et al. 2018	5	90	110	793	-0.92	[-1.84; 0.01]	51.7%	50.6%
Johnson et al. 2015	13	137	7	98	0.28	[-0.67; 1.24]	48.3%	49.4%
**Common effect model**					**-0.34**	**[-1.00; 0.33]**	**100.0%**	**.**
**Random effects model (HK)**					**-0.32**	**[-7.94; 7.30]**	**.**	**100.0%**

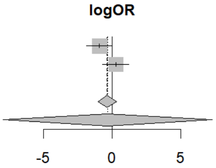

Heterogeneity: $I^2 = 68.1\%$, $\tau^2 = 0.4895$, $\chi^2_1 = 3.13$ ($p = 0.0767$)

Log(OR) and SE:

Log(OR) = -0.322

SE = 0.599

P-value for pooled Log(OR) (random-effects model): 0.686

Study	Cases Exposed	Controls Exposed	Cases Non-Exposed	Controls Non-Exposed	logOR	95%-CI	Weight (common)	Weight (random)
Bhatta et al. 2017	10	101	30	363	0.18	[-0.57; 0.93]	62.5%	62.5%
Johnson et al. 2015	10	100	8	118	0.39	[-0.58; 1.36]	37.5%	37.5%
**Common effect model**					**0.26**	**[-0.33; 0.85]**	**100.0%**	**.**
**Random effects model (HK)**					**0.26**	**[-1.02; 1.54]**	**.**	**100.0%**

Heterogeneity: $I^2 = 0.0\%$, $\tau^2 = 0$, $\chi^2_1 = 0.11$ ($p = 0.7389$)

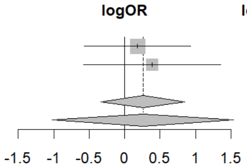

Log(OR) and SE:

Log(OR) = 0.259

SE = 0.101

P-value for pooled Log(OR) (random-effects model): 0.236
