# Supplementary material for: A meta-analysis of the prevalence, genotype distribution and risk factors for human papillomavirus infection in Nepal
Source: PLoS One. 2025 Sep 12;20(9):e0332214. doi: 10.1371/journal.pone.0332214 (PMC12431021; doi:10.1371/journal.pone.0332214)
Supplement: Attachment — Submitted filename: Response to Reviewer.docx [file pone.0332214.s008.docx]

Issue: 1. We note that this manuscript is a systematic review or meta-analysis; our author guidelines require that you upload a PRISMA checklist as supporting information. Information about the PRISMA guidance and blank checklists can be found here: http://www.prisma-statement.org/. Please note that if your study analyzes clinical trials, we cannot proceed with consideration of your article until we have this information.

Response: The PRISMA Checklist is uploaded as supporting information S4 Appendix and S5 Appendix as per journal guideline.
