# Supplementary material for: A meta-analysis of the prevalence, genotype distribution and risk factors for human papillomavirus infection in Nepal
Source: PLoS One. 2025 Sep 12;20(9):e0332214. doi: 10.1371/journal.pone.0332214 (PMC12431021; doi:10.1371/journal.pone.0332214)
Supplement: Attachment — Submitted filename: Response_to_reviewer_auresp_1.docx [file pone.0332214.s009.docx]

**Reviewer #2**

**Abstract**
**Use the full form of ‘human papilloma virus’ for the first instance and use HPV afterward for better clarity (Line: 30)**
Thank you for pointing that out. We have now used the full form "human papillomavirus" at its first mention and followed it with the abbreviation “HPV” in subsequent instances, as suggested.

**Rewrite to better clarity (Line: 39)**
We appreciate your suggestion. The sentence has been revised to enhance clarity.

**Introduction**
**Rewrite “Sexually transmitted infection caused by human papilloma virus (HPV) is common worldwide….” (Line: 43)**
Thank you. The sentence has been rewritten for improved clarity, in line with your recommendation.

**Include references before a full stop throughout the manuscript.**
We have carefully revised the manuscript to ensure that all references are placed before the full stop, as advised.

**“…across various regions of the country” (Line: 51)**
Thank you. We have revised the phrase to “across various regions of the country” for consistency and clarity.

**Line: 52-53, coverage of both vaccination and HPV screening? Should include statistics for this statement.**
Thank you for the suggestion. We have now included statistics from a 2022 study conducted across five tertiary hospitals in Kathmandu, showing HPV vaccination coverage of 1.5% and Pap smear screening at 22.2%, to support the statement.

**Line: 63, use only one ‘strategies’ or ‘policies.’**
We appreciate the feedback. The word “policies” has been removed, and only “strategies” is retained to avoid redundancy.

**Use consistency in tense (Line: 62-75). For example, should write “This review further assessed…” in line 72.**
Thank you for the observation. We have revised the section to ensure consistency in verb tense throughout.

**Line: 77, “generate recommendations”**
The phrase has been updated to “generate recommendations” as per your suggestion.

**Methods**
**Line: 87, why not 31st December 2024? A justification for the timeline should be included.**
Thank you for raising this point. The timeline was set to end on 30th November 2024, as this marked the point at which we completed our literature search and data collection for analysis.

**Line: 94, use abbreviation only**
As recommended, we have replaced “Medical Subject Heading” with its abbreviation “MeSH.”

**Line: 101-115, use bullets or numbering for the inclusion and exclusion criteria**
We have reformatted the inclusion and exclusion criteria using numbering to improve readability.

**“…HPV testing was performed” (Line: 103-104), “…husbands’ migration status” (Line: 107)**
We have made the suggested wording changes accordingly in the specified lines.

**On what basis, individuals were labeled as ‘literate’ or ‘illiterate’? (primary education or anything else) Should be more elaborated (Line: 128-129)**
Thank you for the important observation. We have now clarified that individuals were categorized as “literate” if they had at least primary education or demonstrated the ability to read and write.

**Line: 144, mention ‘(CI)’ for use the abbreviation later**
We have added the abbreviation “(CI)” in the first instance of use, as recommended.

**Results**
**Avoid duplications (Line: 171)**
The duplications in the section have been carefully removed.

**Line: 179, use ‘CI’. Also, use three digits from statistics (CI and p-value) for maintaining consistency.**
We have ensured the use of “CI” and have used three-digit precision for statistics throughout the manuscript to maintain consistency.

**Line: 212-213, what was the p-value?**
Thank you for noticing. We have now added p = 0.33.

**Line: 219, “…among their wives…”**
The phrase has been updated as suggested.

**Rewrite to improve clarity (Line: 221-222)**
We have revised the sentence for better clarity.

**Correct ‘I2” (Line: 231, 233, 235)**
We have corrected the formatting of “I²” in the indicated lines.

**Discussion**
**Avoid mentioning statistics in the discussion section (Line: 254-257, 262-296)**
Thank you. We have revised the relevant paragraphs to remove detailed statistics and focus more on the interpretation of findings.

**Line: 260, “…among women….”**
We have updated the term from “wives” to “women” as suggested.

**Lines: 264-266, discuss comparing with previous findings with reference in a more elaborate way.**
We appreciate this suggestion and have revised the discussion to include more detailed comparisons with previous findings, along with appropriate references.

**Line: 270-271, what are the findings of reviewed articles regarding the importance of vaccination and screening programs? It seems like the authors made assumptions on this, but why did they think so? Please, explain a bit more.**
Thank you for the critical observation. Upon review, we agree that the statement lacked sufficient evidence. We have removed the paragraph to ensure the discussion remains objective and evidence-based.

**Conclusion**
**There was no conclusion section in the manuscript. It seems lines 301-308 are conclusive statements, but the strengths of the study are mentioned afterward. Please rewrite and make necessary rearrangements accordingly.**
We appreciate your feedback. The conclusion section has now been clearly structured and moved to the end of the manuscript, following the strengths and limitations.

**Reviewer #3**

**They were however not able to demonstrate the significance of these factors on the development of HPV infections among Nepal’s women.**
Thank you for this observation. We have now clarified in the Results of Syntheses section that most risk factors did not show statistically significant associations, likely due to limitations such as small sample sizes, heterogeneity, and the cross-sectional nature of the included studies.

**The methodology described was appropriate for the goal of the review but failed to communicate properly the actual steps taken to execute this methodology.**
We appreciate your feedback. We have revised the methodology section to better communicate each step clearly, following PRISMA guidelines.

**While several steps described are reproducible, they were gaps in the presentation that will leave readers feeling lost and confused.**
Thank you for the insight. We have now rewritten the methodology section to ensure greater clarity and coherence for the reader, in alignment with PRISMA standards.

**The statistical analysis and results presentation followed a similar pattern with several steps not clear and their descriptions falling short of acceptable standards in reporting.**
We have revised the descriptions in the Results section to enhance clarity and better align with accepted standards in statistical reporting.

**While the estimation of pooled prevalence was fairly straightforwards, the description of the process and the accompany tables were not self explanatory from the write up.**
Thank you. We have added clearer explanations in the text, and the figure caption now clarifies that the forest plot synthesizes the pooled prevalence while the table details individual study characteristics.

**Similarly, the statistical excursions into the factors associated with HPV infections appeared unnecessary as the data from the reviewed studies do not support such analysis, rendering the conclusions from these analysis void.**
We understand the concern. However, we conducted these analyses only for factors with low heterogeneity and adequate data. We believe these results still contribute meaningfully and have presented them with appropriate caution.

**With such a significant heterogeneity in the studies, the conclusions from these analysis are not supported by the results.**
We acknowledge the limitations due to heterogeneity and have addressed this explicitly in our discussion. Where heterogeneity was high, findings were interpreted cautiously.

**The stated goal to estimate a pooled prevalence is sufficiently weakened by the high degree of heterogeneity.**
We agree that heterogeneity is a known challenge in meta-analyses of observational studies. We have acknowledged this in the discussion and justified the use of a random-effects model accordingly.

**Even though a random effects analysis was done, as stated earlier, the presentation of the analysis was not clear as well as the reporting of the results.**
Thank you. We have worked on improving the clarity of the analysis presentation and reporting of results in the revised manuscript.

**A sensitivity analysis was not reported.**
We appreciate the feedback. Details of the sensitivity analysis have now been included in both the Results section and Supplementary Appendix S6.

**The figures and tables lacked sufficient explanatory notes and were not well presented.**
Thank you for the helpful comment. We have added explanatory captions to all figures and tables to enhance clarity.

**The quality assessment method used was not clear and the table presenting the findings was also not clear.**
We have revised the quality assessment section for better clarity and have reformatted the presentation of the quality appraisal table accordingly.

**The discussion, while acknowledging the limitations of the review, did not sufficiently demonstrated how the authors have met their main goals and objectives.**
Thank you. We have updated the discussion to more clearly link the findings with the objectives of the study.

**They did not show how their findings corroborate with established knowledge on the subjects or differs from regional and global findings.**
We have revised the second and third paragraphs of the discussion to address this point and provide a clearer comparison with regional and global findings.

**The authors also failed to address a number of their specific objectives in both the results and discussion sections.**
We appreciate the feedback. We have now ensured that the objectives—including estimating prevalence, identifying knowledge gaps, and generating recommendations—are clearly addressed in both results and discussion.

**Some of their conclusions were also not supported by the presented analysis and results from this review, especially with respect to risk factors.**
Thank you for the important observation. We have revised the conclusions to be concise, evidence-based, and aligned strictly with our findings. Limitations are now clearly addressed.

**A major review of the manuscript is required to provide clarity, focus and valid conclusions.**
We sincerely thank you for your comprehensive feedback. We have substantially revised the manuscript to improve clarity, maintain focus, and ensure that our conclusions are appropriately supported by the results.
