# Supplementary material for: A meta-analysis of the prevalence, genotype distribution and risk factors for human papillomavirus infection in Nepal
Source: PLoS One. 2025 Sep 12;20(9):e0332214. doi: 10.1371/journal.pone.0332214 (PMC12431021; doi:10.1371/journal.pone.0332214)
Supplement: Attachment — Submitted filename: Response_to_reviewer_auresp_2.docx [file pone.0332214.s010.docx]

We are really thankful to reviewer for insightful comments, which have greatly improved the quality of our manuscript. We hope the revised version now meets the expectations for publication.

Comment: The section of the introduction that describes the goals on this review (Lines 69–78) should be revised to capture only those goals archived by the article.

Response:

We appreciate this observation. The introduction has been revised accordingly. The unaddressed objectives. We now state only the objectives that were actually achieved in the study.

Comment: A major methodological limitation of this article is the number of eligible studies included as well as the type of study design.

Response:

We acknowledge the limited number of eligible studies and the predominance of cross-sectional designs. This limitation has been explicitly mentioned in the “Limitations” subsection of the discussion.

Comment: The results section, while better presented, still lacks sufficient clarity and is difficult to follow. It will benefit from a few tables that adequately capture the relevant results of the analysis, and better description of findings.

We have significantly revised the Results section to improve clarity. Summary tables have been added to present key findings in a reader-friendly format.

Comment: The over-reliance on figures directly exported from statistical software and without appropriate footnotes that explain them undermines the efforts at explaining the results. The reader, if not sufficiently knowledgeable will find it hard to understand this section.

Response:

Only essential figures are retained to enhance readability. Other have been shifter to supplementary file S6_Appendix.

Comment: The authors should present important findings in tables with explanatory summaries under them to enable the reader to appreciate the results being presented.

Response:

As noted above, we have created new summary tables with clear captions and concise summaries below each table to facilitate understanding of key findings

Comment: The discussion section... did not sufficiently discuss the importance of the prevalence reported from this review. The focus on the associated factors (such as multiple sexual partners, contraception, and smoking) took away from the import of the pooled prevalence.

Response:

We have revised the Discussion section to better emphasize the significance of the pooled HPV prevalence. The public health implications of this prevalence in the studied region are now elaborated with supporting references. Discussion of associated factors has been condensed and restructured.

Comment: The literature cited were insufficient and there were implied conclusions that did not exactly correlate with the cited literatures. For example, Line 274–278 links the findings on husband multiple sexual partners and increased risk of HPV infection with high prevalence of HPV DNA and type-specific concordance between infected women and their male partners. While this underscores spousal/partner transmission, it does not suggest husbands have multiple sexual partners.

Response:

Thank you for highlighting this misinterpretation. We have revised the text to clearly distinguish between type-specific concordance and assumptions about partner behavior. The revised text now accurately reflects the cited literature.

Comment: It will be helpful to elaborate this and to cite relevant studies that support the findings on multiple sexual partners. This finding on multiple sexual partners was repeated too frequently in the discussion.

Response:

We have limited the repetition of this point and now discuss it more concisely in a single paragraph with appropriate citations. Redundancies in Lines 295–298 and 323–325 have been removed.

Comment: Line 316 – being the first to attempt a systematic review is not a strength of the systematic review.

Response:

We agree and have removed this sentence. The novelty of our review is now framed more appropriately in terms of its contribution to filling existing gaps in regional HPV prevalence data.

Comment: There is also no need to summarize the methodology again in this section (Lines 319–323). Line 323–325 is a repetition.

Response:

We have deleted the redundant summary of the methodology and the repetitive lines in the Discussion section as suggested.

Comment: The authors should be commended for attempting this systematic review despite the limited number of publications that satisfied the criteria for the review. They have made efforts to compensate for this shortcoming in the various statistical analyses carried out, and have made a fair attempt at presenting and discussing the results.

Response:

We are grateful for this encouraging comment. We have acknowledged the limitations and provided a balanced interpretation of findings in light of these constraints. We are pleased that our efforts have been recognized.
